# Fractional-dose inactivated poliovirus vaccine, India

**DOI:** 10.2471/BLT.18.218370

**Published:** 2019-02-28

**Authors:** Pradeep Haldar, Pankaj Agrawal, Pankaj Bhatnagar, Rajiv Tandon, Sarah McGray, Darin Zehrung, Courtney Jarrahian, Jennifer Foster

**Affiliations:** a Ministry of Health and Family Welfare, New Delhi, India.; b National Polio Surveillance Project, World Health Organization Country Office for India, New Delhi, India.; c PATH, Suite 200, 2201 Westlake Avenue, Seattle, Washington, 98121, United States of America.

## Abstract

In 2016, the World Health Organization (WHO) announced a global shortage of inactivated poliovirus vaccine that was expected to last until 2020 at least. In response, WHO’s Strategic Advisory Group of Experts on Immunization recommended that countries consider a strategic shift to fractional-dose inactivated poliovirus vaccine, which involves a new dosing schedule (i.e. administered at 6 and 14 weeks of age) and has a different mode of delivery than full-dose inactivated poliovirus vaccine (i.e. intradermal rather than intramuscular). Introduction of fractional-dosing requires careful planning and management to ensure adequate vaccine supplies, to prevent wastage, to provide training for health workers, and to ensure accurate record-keeping. In early 2016, given the global vaccine shortage and a limited supply from domestic manufacturers, India’s Expert Advisory Group on polio recommended the staggered introduction of fractional-dosing. India was the first country to introduce fractional-dose inactivated poliovirus vaccine into routine immunization, initially in eight states in 2016. Following a rapid assessment of its initial implementation, fractional-dosing was extended and, by June 2017, all Indian states were covered. Here we summarize India’s experience with the introduction, discuss the challenges faced and the strategies used to address them, and report on the outcomes achieved. We also describe the lessons learnt, especially managing vaccine supplies and wastage, monitoring and supervision, and training needs. As the use of fractional-dose inactivated poliovirus vaccine is dose-sparing and reduces the cost of the immunization programme, it will remain an important part of India’s long-term strategy for polio vaccination.

## Résumé

En 2016, l’Organisation mondiale de la Santé (OMS) a annoncé une pénurie mondiale du vaccin antipoliomyélitique inactivé, prévue pour durer jusqu'à 2020 au minimum. Face à cette situation, le Groupe stratégique consultatif d’experts sur la vaccination a recommandé aux pays d'envisager un changement de stratégie afin de privilégier l’utilisation du vaccin antipoliomyélitique inactivé en doses fractionnées, ce qui implique un nouveau calendrier de vaccination (administration du vaccin à l'âge de 6 et de 14 semaines) et un mode d’administration différent de celui du vaccin antipoliomyélitique inactivé en dose complète (par voie intradermique et non pas par voie intramusculaire). L’introduction d'une vaccination en doses fractionnées exige de la rigueur en matière de planification et de gestion, afin de garantir des stocks de vaccins suffisants, d’éviter les gaspillages, de former les agents de santé et d’assurer une tenue précise des dossiers médicaux. Début 2016, du fait de la pénurie mondiale du vaccin et d’un approvisionnement limité par les fabricants nationaux, le Groupe consultatif d’experts de l’Inde sur l’éradication de la poliomyélite a recommandé d’introduire progressivement les doses fractionnées. Si bien que l’Inde est le premier pays à avoir introduit le vaccin antipoliomyélitique inactivé en doses fractionnées dans le calendrier de vaccination systématique, d’abord dans huit États en 2016. Après une rapide évaluation de cette mise en œuvre initiale, l’utilisation des doses fractionnées s’est étendue, pour finalement être effective dans tous les États indiens en juin 2017. Dans cet article, nous récapitulons l’expérience de l’Inde à ce sujet, nous évoquons les défis rencontrés et les stratégies employées pour les surmonter ainsi que les résultats obtenus. Nous décrivons également les enseignements tirés de cette expérience, notamment en matière de gestion des stocks de vaccins, de prévention des gaspillages, de suivi et de supervision, mais aussi concernant les besoins en formation. Étant donné que l'utilisation de doses fractionnées du vaccin antipoliomyélitique inactivé permet d’économiser des doses vaccinales et de réduire le coût du programme de vaccination, cela restera un élément essentiel dans la stratégie à long terme de l’Inde en matière de vaccination contre la poliomyélite.

## Resumen

En 2016, la Organización Mundial de la Salud (OMS) anunció una escasez mundial de vacunas inactivadas del poliovirus que se esperaba que se prolongara al menos hasta 2020. En respuesta, el Grupo de asesoramiento estratégico de expertos en inmunización de la OMS recomendó que los países consideraran la posibilidad de un cambio estratégico hacia una vacuna inactivada del poliovirus de dosis fraccionada, que incluye un nuevo esquema de dosificación (es decir, administrada a las seis y a las catorce semanas de edad) y que tiene un modo de administración diferente al de la vacuna inactivada del poliovirus de dosis completa (es decir, intradérmica y no intramuscular). La introducción de la dosis fraccionada requiere una planificación y una gestión minuciosas para garantizar el suministro adecuado de las vacunas, evitar el despilfarro, formar a los trabajadores sanitarios y garantizar el mantenimiento de registros precisos. A principios de 2016, dada la escasez mundial de vacunas y el limitado suministro de los fabricantes nacionales, el Grupo de asesoramiento experto sobre la polio de la India recomendó la introducción escalonada de dosis fraccionadas. La India fue el primer país en introducir la vacuna inactivada del poliovirus de dosis fraccionada en la inmunización sistemática, inicialmente en ocho estados en 2016. Tras una rápida evaluación de la aplicación inicial, se amplió la dosificación fraccionada y, para junio de 2017, se cubrieron todos los estados de la India. En este documento se resume la experiencia de la India con la introducción, se examinan los problemas encontrados y las estrategias utilizadas para resolverlos y se informa sobre los resultados alcanzados. También se describen las lecciones aprendidas, especialmente en lo que se refiere a la gestión de los suministros de vacunas y el desperdicio, el seguimiento y la supervisión, y las necesidades de formación. Dado que el uso de la vacuna inactivada del poliovirus de dosis fraccionada ahorra dosis y reduce el coste del programa de inmunización, seguirá siendo una parte importante de la estrategia a largo plazo de la India para la vacunación contra la polio.

## ملخص

في عام 2016، أعلنت منظمة الصحة العالمية ( **WHO ** ) عن نقص عالمي في لقاح فيروس شلل الأطفال غير النشط، والذي كان من المتوقع أن يستمر حتى عام 2020 على الأقل. وردا على ذلك، أوصت مجموعة الخبراء الاستشاريين الاستراتيجية المعنية بالتحصين، والتابعة لمنظمة الصحة العالمية بأن تدرس البلدان التحول الاستراتيجي إلى لقاح غير نشط لشلل الأطفال بجرعة جزئية، والذي يتضمن جدول جرعات جديد (أي يُعطى في عمر 6 أسابيع و 14 أسبوعًا) ولديه طريقة مختلفة للحقن غير الجرعة الكاملة النشطة من لقاح شلل الأطفال (أي داخل الأدمة وليس داخل العضل). يتطلب حقن الجرعات الجزئية التخطيط والإدارة بعناية لضمان توفير إمدادات كافية من اللقاحات، لمنع الفاقد من توفير التدريب للعاملين في القطاع الصحي، وضمان الاحتفاظ بسجلات دقيقة. في أوائل عام 2016، وفي ظل النقص العالمي في اللقاح، والإمدادات المحدودة من جهات التحضير المحلية، أوصت مجموعة الخبراء الاستشاريين الهندية المعنية بشلل الأطفال بإدخال جرعات جزئية متداخلة. كانت الهند أول بلد يدخل جرعة لقاح شلل الأطفال غير النشطة جزئيا في التحصين الروتيني، ومبدئياً في ثماني ولايات في عام 2016. وبعد إجراء تقييم سريع للتنفيذ المبدئي، تم توسيع الجرعات الجزئية، وبحلول يونيو/حزيران 2017، تم تغطية جميع الولايات الهندية. نحن نوجز هنا تجربة الهند في طرح الفكرة، ونناقش التحديات التي تواجهها والاستراتيجيات المستخدمة للتعامل معها، والإبلاغ عن النتائج المحققة. كما نصف الدروس المستفادة، لاسيما إدارة إمدادات اللقاحات والإهدار والرصد والإشراف، واحتياجات التدريب. وبما أن استخدام لقاح فيروس شلل الأطفال غير النشط بجرعة جزئية هو عملية ترشيد للجرعة، ويقلل من تكاليف برنامج التحصين، فإنه سيظل جزءًا هامًا من استراتيجية الهند طويلة المدى للتطعيم ضد شلل الأطفال.

## 摘要

2016 年，世界卫生组织 (WHO) 宣布灭活脊髓灰质炎疫苗面临全球范围内的短缺，此情况预计将至少持续至 2020 年。为了应对此情况，世卫组织免疫接种战略咨询专家组建议各国将分剂量灭活脊髓灰质炎疫苗的战略性转变纳入考量，这涉及新的给药方案（即在 6 和 14 周时给药）并具有不同于全剂量灭活脊髓灰质炎疫苗的注射方式（即皮内注射而非肌肉注射）。引入分剂量注射方式需要细致规划和管理，从而保证疫苗供应充足、避免损耗，对医务工作者进行培训，确保精准记录。2016 年初，鉴于全球疫苗短缺以及国内疫苗制造商供应不足，印度脊髓灰质炎专家咨询小组建议错开分剂量灭活脊髓灰质炎疫苗的接种时间。印度是第一个将分剂量灭活脊髓灰质炎疫苗纳入常规免疫接种的国家，并于 2016 年开始在 8 个邦实施接种。对首次实施分剂量疫苗接种进行快速评估后，印度迅速扩大了分剂量灭活脊髓灰质炎疫苗的接种范围。截至 2017 年 6 月，印度各邦均已覆盖。本文归纳总结了印度在接种分剂量疫苗方面的经验，对其面临的挑战以及应对策略予以讨论并汇报了所取得的成果。我们还对以往的经验教训进行了回顾总结，尤其是在疫苗的供应、损耗管理方面、监测与监督方面以及培训需求方面。由于分剂量灭活脊髓灰质炎疫苗的使用可以节省剂量并降低免疫计划的成本，因此它将成为印度脊髓灰质炎疫苗接种长期战略计划的重要组成部分。

## Резюме

В 2016 году Всемирная организация здравоохранения (ВОЗ) объявила о глобальной нехватке инактивированной полиовакцины, которая может продлиться как минимум до 2020 года. В ответ на это Стратегическая консультативная группа экспертов ВОЗ по иммунизации рекомендовала странам рассмотреть стратегию перехода на частичную дозировку инактивированной полиовакцины, что подразумевает новый режим дозирования (в 6 и 14 недель после рождения) и иной способ введения (внутрикожно, а не внутримышечно, как это делалось в случае введения полноценной дозы инактивированной полиовакцины). Переход на частичную дозировку требует тщательного планирования и отслеживания, чтобы обеспечить достаточные поставки вакцины, предотвратить потери, обучить медицинских работников, а также гарантировать точное ведение документации. В начале 2016 года, ввиду глобальной нехватки вакцины и ограниченных поставок от отечественных производителей, Консультативная группа экспертов Индии по полиомиелиту рекомендовала поэтапный переход на частичную дозировку. Индия стала первой страной, внедрившей инактивированную полиовакцину в частичной дозировке в график плановых мероприятий по иммунизации изначально в восьми штатах в 2016 году. На основании оперативной оценки начального этапа перехода было принято решение расширить использование частичной дозировки, и к июню 2017 года все штаты Индии перешли на использование новой схемы. В статье приведен краткий обзор опыта Индии по переходу на новую схему, обсуждаются возникшие проблемы и стратегии, используемые для их решения, а также представлен отчет о достигнутых результатах. Авторы описывают полученный опыт, в частности мероприятия по управлению поставками вакцины и предотвращению потерь, мониторингу и надзору, а также потребности в области профессиональной подготовки. Поскольку использование инактивированной полиовакцины в частичной дозировке позволяет экономить дозы и сокращает стоимость программы иммунизации, данный подход станет неотъемлемой частью долговременной программы Индии по вакцинации от полиомиелита.

## Introduction

In early 2016, the World Health Organization (WHO) announced a global shortage of inactivated poliovirus vaccine,^1^^,^^2^ which was estimated in 2017 to last until 2020 and potentially beyond.^3^ In response, WHO’s Strategic Advisory Group of Experts on Immunization recommended that countries with good immunization systems and coverage consider administering two fractional inactivated poliovirus vaccine doses of 0.1 mL each intradermally instead of a single, intramuscular, full dose of 0.5 mL.^4^^,^^5^

The use of fractional doses has important implications for immunization systems, including the supply chain. When a vial intended for full-dose vaccination is used to deliver fractional doses, the number of doses it contains increases by a factor of five: a vial containing 5 or 10 full doses becomes a 25- or 50-fractional-dose vial, respectively. Moreover, as the number of doses available at a facility increases, the supply frequency must change to accommodate the slower consumption of individual vials. Staff must undergo training on procedures for new vaccine delivery schedules and on the different route of administration. Vaccinators may, therefore, need to be refamiliarized with the Mantoux method. Finally, close monitoring is essential to ensure that injection quality, vaccine wastage and supplies of both vaccine and injection equipment are appropriate and that coverage meets set targets.

India was the first country in the world to introduce fractional-dose inactivated poliovirus vaccine into its immunization programme. A phased introduction started in 2016 and coverage was expanded throughout the country in 2017. This paper describes the background to the introduction of fractional-dosing in India and highlights aspects of the country’s immunization programme that were critical for success. We provide information about immunization programme features, such as training health-care workers, monitoring the introduction of fractional-dosing and vaccine usage, and updating vaccine supply and distribution plans, that could be useful for other countries considering the use of fractional-dose inactivated poliovirus vaccine.

## Introducing fractional-dosing

**Fig. 1:**
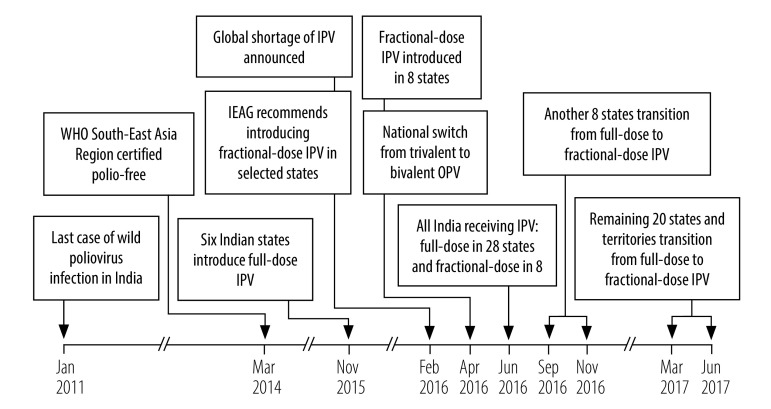
Timeline for the introduction of inactivated poliovirus vaccine and fractional-dose administration, India, 2016–2017

IEAG: India Expert Advisory Group; IPV: inactivated poliovirus vaccine; OPV: oral poliovirus vaccine; WHO: World Health Organization.

Following publication of the global Polio Eradication Endgame and Strategic Plan 2013–2018 and of recommendations by WHO’s Strategic Advisory Group of Experts on Immunization,^6^^,^^7^ the Indian government decided to introduce inactivated poliovirus vaccine into its routine immunization programme in preparation for the planned global switch from trivalent to bivalent oral poliovirus vaccine in April 2016. For the first year, the government requested 40 million doses of inactivated poliovirus vaccine from Gavi, the Vaccine Alliance. However, Gavi agreed to supply only 28 million doses, which was insufficient to cover the full birth cohort. Consequently, the government decided to phase in the introduction of inactivated poliovirus vaccine and full-dose vaccination began in six states in November 2015 (Fig. 1). To supplement vaccines supplied by Gavi, the government started to procure vaccines using its domestic budget. Since the first quarter of 2017, all inactivated poliovirus vaccines used in India has been paid for by the Indian government. However, due to global shortages, domestic manufacturers were not able to provide an adequate supply despite their best efforts.

Even with the staggered introduction of inactivated poliovirus vaccine in India, supply challenges remained. In February 2016, the India Expert Advisory Group on polio convened an interim meeting to discuss the impact of supply constraints on the scheduled switch to bivalent oral poliovirus vaccine in April that year.^8^ Based on the evidence that two fractional inactivated poliovirus vaccine doses yields equivalent or greater immunogenicity as a single full inactivated poliovirus vaccine dose,^5^^,^^9^^–^^11^ the advisory group recommended that the government continue with the planned switch to bivalent oral poliovirus vaccine and consider implementing a routine immunization schedule of two fractional doses, at 6 and 14 weeks of age, as a risk-mitigation strategy. The advisory group also recommended that fractional-dose vaccination be initially introduced in selected states and, depending on operational feasibility and experience with the programme, then be expanded to other states.^8^ Following these recommendations, fractional-dosing was introduced in eight selected states, while the remaining states used full doses.

In April 2016, Indian states and territories fell into one of three categories: (i) states that had already started using full-dose vaccine; (ii) states and territories that would introduce fractional-dose vaccine; and (iii) states and territories that would continue with the introduction of full-dose vaccine and switch to fractional-dose vaccine at a later date. By June 2016, some form of the vaccine was being used across the entire country: 28 states and territories were using full doses and 8 were using fractional doses.

A rapid assessment of the introduction of fractional-dosing, conducted by WHO’s country office in India in partnership with the Indian government, showed that its initial introduction in eight states was successful and could be replicated in other states. This assessment also identified operational areas that could be improved, such as: (i) the display of information, education and communication materials; (ii) the identification of children eligible for vaccination; and (iii) vial use and storage (some cold chain handlers were unaware that the shake test is not applicable to inactivated poliovirus vaccine and that vials should not be kept in ice packs because of the risk of vaccine damage by freezing). Based on this assessment, the decision was made to switch states that were still using full inactivated poliovirus vaccine doses over to fractional doses. The subsequent transition from full to fractional doses was staggered; 8 further states transitioned in September 2016 and the remaining 20 transitioned in the first two quarters of 2017 (Fig. 1). In June 2016, fractional-dose vaccine was used in a campaign in Telangana State in response to vaccine-derived poliovirus type 2 being detected in sewage.^12^

## Introduction challenges

In early 2016, Indian states and territories either had already started using full doses of inactivated poliovirus vaccine, were planning to introduce fractional doses or were planning to introduce full doses and switch to fractional doses later. Consequently, careful planning was required and different strategies were adopted to deal with: (i) advocacy and training; (ii) monitoring the introduction and use of inactivated poliovirus vaccine (both full and fractional doses); and (iii) managing the supply of vaccine to ensure its distribution was aligned with the varying number of children served at individual clinics, thereby preventing wastage. The new dosing schedule, the new mode of vaccination and the increased number of doses per vial all presented challenges. However, due to the global shortages, several steps were already being taken in India to ensure a smooth vaccine supply before fractional-dosing was introduced. These measures continue to support the polio immunization programme.

### Initial preparations

There was very little time between the decision to use fractional-dosing (i.e. in February 2016) and its planned implementation, which was to take place as soon as possible because type-2-containing oral poliovirus vaccine was scheduled to be withdrawn in April 2016. During this short interval, information, education and communication materials were modified and staff, who had already received training to use full-dose inactivated poliovirus vaccine, were retrained. In addition, state governments were provided with information on, and support with, the introduction of fractional-dose inactivated poliovirus vaccine, which is an off-label use. The decision to introduce fractional-dosing initially in only eight selected states meant that advocacy and training activities could be targeted. The selection of states also provided an opportunity to assess its introduction in a pilot programme. Since inactivated poliovirus vaccine had already been approved in India for full-dose use, the recommendations made by the India Expert Advisory Group enabled off-label use of the vaccine in a fractional-dose schedule to pass quickly through the necessary regulatory processes.

States that were transitioning from full-dose to fractional-dose vaccine from September 2016 onwards were allowed a buffer period of 3 months. During this time, health-care facilities were instructed to use fractional doses for children receiving inactivated poliovirus vaccine for the first time at 6 weeks of age, they were treated again at 14 weeks of age. Children older than 6 weeks who had not previously received the vaccine were given a single full dose at 14 weeks of age. After this buffer period, all children received fractional-dose inactivated poliovirus vaccine at 6 and 14 weeks of age. For health-care workers to understand the change in vaccination schedule, training materials were modified in states switching to fractional-dose inactivated poliovirus vaccine.

The phased introduction of inactivated poliovirus vaccine meant that dosing schedules and modes of vaccine delivery varied across different states, both within clinics during the buffer period and between public and private sector facilities. This posed challenges for recording and reporting. Accurate dose-tracking was particularly difficult as people are mobile and often migrate from place to place. Monthly reports were adapted to track the use of both full- and fractional-dose vaccine.

### Forecasting vaccine demand

In 2016 and 2017, the total demand for inactivated poliovirus vaccine increased even as the projected demand decreased in states transitioning to fractional-dose inactivated poliovirus vaccine, principally because full-dose inactivated poliovirus vaccine was simultaneously being extended to other states. Vaccine supplies and variations in consumption were carefully monitored at the national level to ensure that the appropriate quantity was distributed to each state. Vaccines were distributed according to standard guidelines (i.e. first expiry, first out and first in, first out). In addition, the national immunization programme asked suppliers to adjust the supply schedule to avoid stock shortages and surpluses.

India also began using a consumption-based approach to supplying inactivated poliovirus vaccine to facilities: the number of doses delivered in any month was the number delivered in the previous month plus 10%. This contrasted with the projection-based approach used for other routine immunizations. The result was that national immunization programme supervisors were able to closely track vaccine consumption patterns in each clinic, including the mix of full- and fractional-dose vaccine during and after the 3-month buffer period, when both were used. However, this approach meant that a facility that underperformed in a given month could receive fewer vaccine doses in the following month, which made it difficult to meet any rise in demand for vaccination coverage. To address this concern, the national immunization programme increased the proportion of additional doses supplied to 20% and continued to monitor use closely. Also, states were instructed to raise their interim demand for vaccine as needed, so long as they submitted a report on the vaccines delivered.

In October 2015, India established an electronic Vaccine Intelligence Network, which is a digital system for real-time monitoring of the supply chain that has proven helpful in maintaining stocks of vaccine at recommended temperatures.^13^ The network was initially introduced in 12 states, where it covered around 10 500 of the existing 27 000 cold chain points and served nearly 60% of children younger than 2 years.

### Preventing vaccine wastage

As the national immunization programme closely monitored consumption of inactivated poliovirus vaccine at the clinic level, the programme was well positioned to rapidly identify supply shortages and variations in wastage. In India, there are substantial intra- and inter-state variations in population density (70% of the country’s birth cohort is concentrated in 10 of the 29 states) and consumption projections for densely populated states are inappropriate for sparsely populated states. Early during the introduction of inactivated poliovirus vaccine, when both vials containing 5 and 10 full doses were in the supply chain, it was anticipated that facilities serving low-density populations (e.g. in hilly, tribal and remote areas) would experience higher wastage because they would be unable to completely use a multidose vial within 28 days, when open vials must be discarded. In particular, there was a concern that high wastage would outweigh the benefits of the dose-sparing fractional-dose schedule. In areas with a sparse population, the estimated wastage for fractional doses taken from a 10-full-dose vial was 60%, compared with 20% with a 5-full-dose vial. For comparison, the estimated wastage for full-dose vaccination using a 10-dose vial was 10%.

As smaller vaccine vials helped minimize wastage, states using fractional-dosing and areas that had a low population density or were difficult to reach were supplied with vials containing five full doses. In addition, states were also asked to plan vaccine distribution to minimize wastage while strictly implementing the policy of discarding opened vials within 28 days. They were also reminded that the shake test is not applicable to inactivated poliovirus vaccine. States submitted their monthly consumption reports manually because, in 2017, inactivated poliovirus vaccine was not included in the online health management information system for reporting coverage data. The distribution of vaccine to states was kept under national control.

Furthermore, India requested and received support from the Global Polio Eradication Initiative through the United Nations Children's Fund to convert 2 million inactivated poliovirus vaccine doses provided through Gavi for the first year from 10-full-dose vials to a 5-full-dose format. All domestic supplies are in 5-full-dose vials.

As an early adopter of fractional-dosing, India set new norms for adjusting existing inactivated poliovirus vaccine policy and practices to accommodate the unique requirements of transitioning from full-dose to fractional-dose vaccine. Successful implementation relied on careful planning at the outset to consider the individual requirements of the large variety of immunization settings in the country. Estimating wastage before the transition was critical for identifying where fractional-dose vaccine could be best applied. In addition, vigilant monitoring of monthly reports ensured that changes in vaccine supply, demand or committed quantities could be accommodated and enabled the rapid identification of places where refresher training may be required.

## Outcomes

**Fig. 2:**
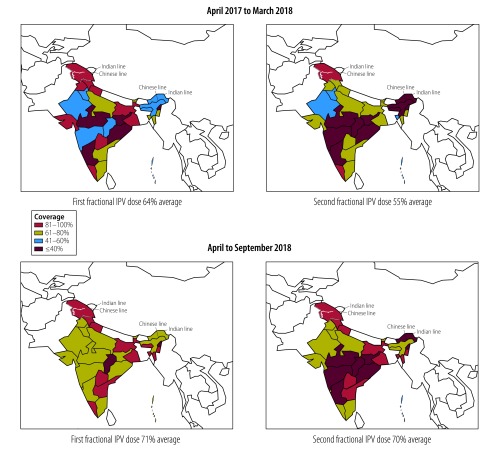
Coverage of fractional-dose inactivated poliovirus vaccine, by dose and state, India, April 2017 to September 2018

**Fig. 3:**
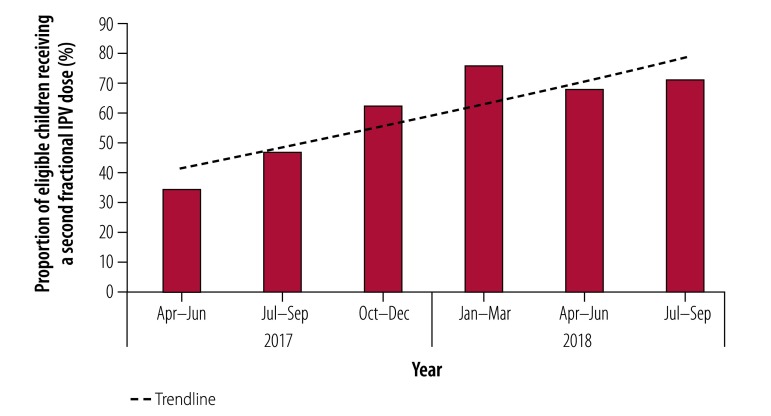
National coverage of fractional-dose inactivated poliovirus vaccine, India, April 2017 to September 2018

India began the staggered introduction of full-dose inactivated poliovirus vaccine in November 2015 and, by June 2016, the entire country was receiving either full-dose or fractional-dose vaccine. By June 2017, all Indian states had transitioned to using two fractional vaccine doses (Fig. 2). No vaccine-derived poliovirus type-2 cases have been detected since the adoption of fractional-dosing. In addition to making policy changes to facilitate the introduction of fractional-dose vaccine, the Indian government has also made efforts to rapidly increase immunization coverage through special immunization drives under the Mission Indradhanush programme, whose aim was to achieve full immunization coverage (i.e. greater than 90%) by December 2018. This programme, which focuses on children younger than 2 years and pregnant women, has helped strengthen the overall immunization system and increase coverage. The close tracking and immunization of eligible recipients of inactivated poliovirus vaccine combined with the more appropriate distribution of vaccines has contributed to a rapid increase in the coverage of fractional-dose inactivated poliovirus vaccine, which reached 71 % by September 2018 (Fig. 3) according to information from Indian states (Indian health management information system, unpublished data, 2017).

IPV: inactivated poliovirus vaccine.

Note: The figure shows coverage compared to targets.

IPV: inactivated poliovirus vaccine.

Notes: The figure shows coverage compared to targets. Data were obtained from reports submitted manually by Indian states and from the Indian health management information system. The data for April to June 2018 were provisional.

## Lessons learnt

The key lessons learnt from the introduction of fractional-dose inactivated poliovirus vaccine in India relate to: (i) strategies addressing vaccine supply and wastage; (ii) managing the introduction of fractional-dose vaccine; (iii) managing the transition from full-dose to fractional-dose vaccine; and (iv) communicating and coordinating with stakeholders. These lessons informed the introduction of fractional-dose inactivated poliovirus vaccine in Sri Lanka.^14^

In settings where there are few potential beneficiaries, vaccine wastage should be minimized by using vials containing fewer doses, perhaps 1 or 2 full doses. Smaller vials would also allow greater flexibility in planning immunization sessions in sparsely populated areas. Procurement frequency and supply quantities must be adjusted to match the slower drain on vaccine supplies that results from using fractional rather than full doses. In addition, regular consultations with regulatory authorities, suppliers, states and partners are essential for ensuring the appropriate regulation, procurement and distribution of vaccine supplies.

Close monitoring and supportive supervision are critical for rapidly identifying and remediating any problems with the introduction of fractional-dosing. Clinic records should be reviewed regularly to ensure adherence to recommended dosing schedules and dose volumes and periodic spot checks should be carried out to ensure the correct injection technique is being applied. Clear guidance should be given on how to determine whether a full or fractional-dose should be given to an individual after the introductory buffer period in settings where a transition from full-dose to fractional-dose vaccine has been planned. In addition, records for the Expanded Programme on Immunization must be updated before fractional-dose inactivated poliovirus vaccine is introduced to ensure that dose-tracking is accurate, particularly during the buffer period when both full and fractional-doses are being used.

Health-care workers in India are familiar with intradermal injection using the Mantoux method because they routinely give Bacillus Calmette-Guérin vaccine for tuberculosis. Therefore, they required no additional training on intradermal injection techniques. However, where this is not the case, refresher courses on the correct injection technique should be provided while health-care workers and Expanded Programme on Immunization managers are undergoing training on the new vaccination schedule, dose volume and supply frequency required for fractional-dose inactivated poliovirus vaccine.

Although India has successfully introduced fractional-dose inactivated poliovirus vaccine in all states, there are several future challenges. Since the initial support provided by Gavi and the Global Polio Eradication Initiative ended in 2016, India has been procuring inactivated poliovirus vaccine using the domestic budget. However, the most recent quote from the only domestic manufacturer was 80% higher than the previous price, 179.59 Indian rupees (i.e. 2.64 United States dollars) per dose. This increase has put India in a difficult position because paying more for inactivated poliovirus vaccine requires a trade-off with other public health priorities, such as expanding rotavirus and pneumococcal vaccination and strengthening the overall immunization programme. An India Expert Advisory Group meeting on polio eradication held in June 2018 recommended that the country should continue to include inactivated poliovirus vaccine in its routine immunization programme. Accordingly, India proceeded with procuring the vaccine to support global polio eradication. The nationwide expansion of fractional-dose vaccine, which requires 0.2 mL of vaccine per child rather than the 0.5 mL needed for full-dose vaccination, has been cost–effective and has contributed to vaccine security, both nationally and globally, because supplies are likely to remain tight until 2020. Consequently, fractional-dosing remains an important part of the long-term polio vaccination strategy in India.

In conclusion, the challenges of transitioning from full-dose to fractional-dose inactivated poliovirus vaccine in India may not be the same as in other nations because India has the largest birth cohort globally and a strong vaccine supply chain. However, the lessons learnt should be applicable to most countries, regardless of their size or the strength of their immunization programme. We hope our experience will provide a foundation for planning and monitoring the introduction of fractional-dose inactivated poliovirus vaccine. If the availability and cost of inactivated poliovirus vaccine remain a concern, the use of fractional doses can reduce the cost of good coverage and thus support the objective of polio eradication.
